# T Cell Detection of a B-Cell Tropic Virus Infection: Newly-Synthesised versus Mature Viral Proteins as Antigen Sources for CD4 and CD8 Epitope Display

**DOI:** 10.1371/journal.ppat.1000699

**Published:** 2009-12-18

**Authors:** Laura K. Mackay, Heather M. Long, Jill M. Brooks, Graham S. Taylor, Carol S. Leung, Adrienne Chen, Fred Wang, Alan B. Rickinson

**Affiliations:** 1 School of Cancer Sciences and MRC Centre for Immune Regulation, College of Medicine, University of Birmingham, Birmingham, United Kingdom; 2 Department of Medicine, Brigham & Women's Hospital, Harvard Medical School, Boston, Massachusetts, United States of America; University of Wisconsin-Madison, United States of America

## Abstract

Viruses that naturally infect cells expressing both MHC I and MHC II molecules render themselves potentially visible to both CD8^+^ and CD4^+^ T cells through the *de novo* expression of viral antigens. Here we use one such pathogen, the B-lymphotropic Epstein-Barr virus (EBV), to examine the kinetics of these processes in the virally-infected cell, comparing newly synthesised polypeptides versus the mature protein pool as viral antigen sources for MHC I- and MHC II-restricted presentation. EBV-transformed B cell lines were established in which the expression of two cognate EBV antigens, EBNA1 and EBNA3B, could be induced and then completely suppressed by doxycycline-regulation. These cells were used as targets for CD8^+^ and CD4^+^ T cell clones to a range of EBNA1 and EBNA3B epitopes. For both antigens, when synthesis was induced, CD8 epitope display rose quickly to near maximum within 24 h, well before steady state levels of mature protein had been reached, whereas CD4 epitope presentation was delayed by 36–48 h and rose only slowly thereafter. When antigen expression was suppressed, despite the persistence of mature protein, CD8 epitope display fell rapidly at rates similar to that seen for the MHC I/epitope half-life in peptide pulse-chase experiments. By contrast, CD4 epitope display persisted for many days and, following peptide stripping, recovered well on cells in the absence of new antigen synthesis. We infer that, in virally-infected MHC I/II-positive cells, newly-synthesised polypeptides are the dominant source of antigen feeding the MHC I pathway, whereas the MHC II pathway is fed by the mature protein pool. Hence, newly-infected cells are rapidly visible only to the CD8 response; by contrast, latent infections, in which viral gene expression has been extinguished yet viral proteins persist, will remain visible to CD4^+^ T cells.

## Author Summary

Many viruses infect cells in which both the MHC I and MHC II pathways of antigen presentation are active, and so viral proteins expressed in those cells may be presented as MHC I-peptide complexes to CD8^+^ T cells and as MHC II-peptide complexes to CD4^+^ T cells. Here we study these processes in a model system involving Epstein-Barr virus-infected human B lymphocytes (MHC I/II-positive) where viral antigen expression can be induced or suppressed at will, and antigen presentation tracked with specific CD8^+^ and CD4^+^ T cell clones. In this system, we find that the MHC I pathway is entirely fed by newly-synthesised polypeptides, whereas the MHC II pathway depends upon antigen supplied from the mature protein pool. Hence, while only CD8^+^ T cells can rapidly recognise new infections, only CD4^+^ T cells will recognise latent infections in which viral gene expression is extinguished yet a pool of viral antigens remains.

## Introduction

Many intracellular pathogens, particularly viruses, naturally infect cells of the haemopoietic system that express both MHC I and MHC II molecules. Such infected cells may be rendered visible to the host T cell response through the intracellular processing of virally-encoded proteins, leading to cell surface display of MHC I- and MHC II- peptide complexes recognised by CD8^+^ and CD4^+^ T cells respectively. With regard to MHC I-restricted presentation, the speed with which virus-infected cells become recognisable by CD8^+^ T cells [1] and the involvement of the proteasome in that process [2] led to the idea that a proportion of all newly-synthesised viral polypeptides were marked for immediate degradation, generating peptides that were fed into the MHC I pathway [3]. While the concept has evidential support [4],[5],[6],[7], questions remain about the proportion of translation products sacrificed in this way [8],[9], the mechanism that underpins their selection [10],[11] and most importantly the degree to which, in latently-infected cells where viral antigen synthesis has been extinguished, cells may still be visible to the virus-specific CD8 response through MHC I-restricted processing of antigen from the mature protein pool. Only two studies have attempted to address this latter issue by specifically regulating antigen expression rather than resorting to general inhibitors of translation [12],[13]. Though both studies supported the dominance of newly-synthesised protein as an antigen source, in each case the evidence came from a single epitope studied at a very limited number of time points leaving the generality of the results, with respect to such variables as antigen dose, epitope location and target cell identity, unresolved.

Less is known about the rules governing MHC II-restricted presentation of endogenously expressed viral antigens, though it is clear that under some circumstances this can occur [14],[15]. To date there are examples of endogenous antigen accessing the MHC II pathway either through location in the endoplasmic reticulum itself [16], through delivery to endosomes/lysosomes by macro- [17],[18] or chaperone-mediated [19] autophagy, or through release and re-uptake by neighbouring cells [20]. However there is little information on two important issues: firstly the kinetics with which MHC II-restricted epitopes are presented following antigen expression, which determines when a newly-infected cell becomes visible to the CD4^+^ T cell response, and secondly the relative importance of newly-synthesised polypeptides and the mature protein pool as antigen sources.

Here we address these issues using Epstein-Barr virus (EBV), a human gamma-herpesvirus that transforms B cells *in vitro* into MHC I/II-positive lymphoblastoid cell lines (LCLs) expressing eight viral proteins, the nuclear antigens EBNAs 1, 2 3A, 3B, 3C and -LP, and the latent membrane proteins LMPs 1 and 2 [21]. Such LCLs resemble the virus-transformed B cells that arise during EBV infection *in vivo* and elicit the MHC I- and MHC II-restricted T cell responses that control the infection [22]. Many of these responses have been mapped to individual peptide epitopes and epitope-specific CD4^+^ and CD8^+^ T cell clones shown to recognise MHC-matched LCL targets [22]. Here we sought to use such clones to follow the presentation of EBV antigens via the MHC I and MHC II pathways in an LCL background which lacked base-line epitope display and where expression of the cognate antigen could be temporally controlled. For this purpose we chose two indicator antigens, EBNA3B and EBNA1. EBNA3B is non-essential for transformation *in vitro* and therefore one can establish LCLs with an EBNA3B gene-deleted virus [23],[24]; EBNA1, the virus genome maintenance protein, is required for transformation but shows sequence variation between virus isolates, allowing one to establish LCLs using a virus that lacks many of the relevant T cell epitopes [25],[26]. In both cases we then introduced the cognate antigen-coding sequence under the control of a doxycycline-regulated promoter and monitored CD4 and CD8 epitope display after inducing or suppressing new antigen synthesis.

## Results

### Characterisation of the dox-regulated expression system using EBNA3B

**Figure 1:**
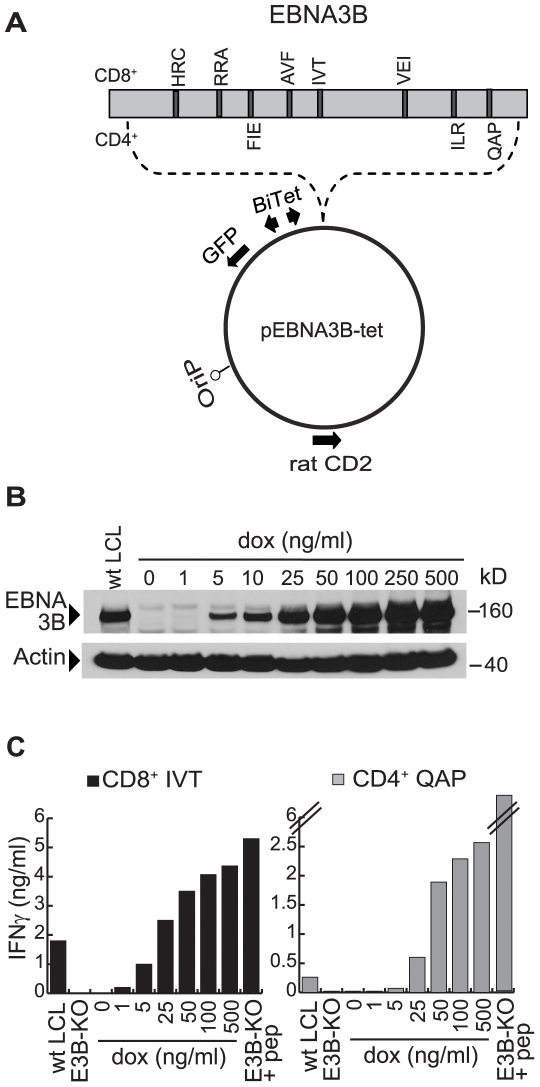
Inducible expression of EBNA3B as an indicator antigen. (A) Map of the pEBNA3B-tet vector, a derivative of the dox-dependent expression vector pRTS-1 [27]. Rat CD2 (expressed as a truncated signalling-negative surface protein) allows for selection post-transfection. oriP is the EBV origin of plasmid replication and mediates long-term maintenance of the vector as an episome in LCL cells. A bidirectional dox-regulated promoter (BiTet) controls expression of the gene insert and GFP. Here the gene insert encodes EBNA3B (grey box); T cell epitopes are located by dark lines and identified above (CD8) or below (CD4) by three letter code. (B) Immunoblot showing EBNA3B protein levels in a pEBNA3B-tet LCL 7 days after dox-induction at 0–500 ng/ml; EBNA3B as expressed in a wild-type B95.8 virus-transformed LCL (wt LCL) is shown for comparison. Actin levels serve as a loading control. (C) 7 days post-induction, the above pEBNA3B-tet LCL cells (A*1101, DRB3*0201-positive) were fixed and used as targets for CD8^+^ and CD4^+^ T cell clones specific for the EBNA3B-derived IVT/A*1101 and QAP/DRB3*0201 epitopes respectively. Recognition (mean of triplicate values +/− SD) is expressed as IFNγ units/ml detected in assay supernatant by ELISA. Reference targets, all from the same donor as above, were a wt LCL and an EBNA3B-KO virus-transformed LCL (E3B-KO) with and without pre-exposure to the relevant epitope peptide at 10^−6^ M. Results are representative of three experiments. Assays involving two further pEBNA3B-tet LCLs, CD8^+^ clones to four other epitopes and CD4^+^ clones to two other epitopes gave a similar pattern of results.

Figure 1A shows the vector used to achieve dox-dependent antigen expression [27]. Rat CD2 expression from the vector backbone allows initial enrichment of transfected cells, while the EBV *ori-p* sequence promotes episomal maintenance in LCLs. Antigen-coding sequences lie under the control of a dox-regulated promoter. We first introduced an EBNA3B-carrying vector (pEBNA3B-tet, Figure 1A) into LCLs made using a recombinant EBNA3B-KO virus. Figure 1B illustrates the pattern of results consistently observed with stable pEBNA3B-tet transfectants on three different LCL backgrounds. EBNA3B protein expression, undetectable by immunoblotting in non-induced cells, showed a clear dose-dependent response to 7 day treatment with dox, reaching a level equivalent to that seen in wild-type EBV-transformed LCLs at 25 ng/ml dox and increasing to supra-physiologic levels at higher dox concentrations.

We then assayed these same cells, dox-induced for 7 days, as targets for T cell recognition. CD8^+^ T cell clones were generated against five well-defined epitopes in EBNA3B (HRC/B*2705, RRA/B*2702; AVF/A*1101, IVT/A*1101, VEI/B*4402; positions shown in Figure 1A, see Table S1 for details). Because EBNA3B had not been studied before as a CD4 target, we first screened EBV-immune donors for CD4^+^ T cell reactivity to an EBNA3B peptide panel in IFNγ Elispot assays, generated CD4^+^ T cell clones against three of the epitopes thus defined and determined their MHC II restriction using standard approaches [28],[29]. These epitopes (FIE/DRB1*1501, ILR/DRB4*01 and QAP/DRB3*0201; see Table S1) are located on the EBNA3B sequence in Figure 1A. Figure 1C shows representative results from such experiments, here using a pEBNA3B-tet LCL (A*1101/DRB3*0201-positive) as a target for CD8^+^ clones against the IVT/A*1101 epitope and for CD4^+^ clones against the QAP/DRB3*0201 epitope. All such experiments included, as a positive control target, a wild-type EBV-transformed LCL from the same individual expressing EBNA3B from the resident EBV genome. Target cell recognition is assayed by IFNγ release after 18 h of co-culture. There was no response to the non-induced pEBNA3B-tet LCL by either CD8^+^ or CD4^+^ effectors, whereas dox-induced cells were recognised at levels which increased in a dose-dependent manner. For both effector populations, the recognition of target cells exposed to 25 ng/ml dox (i.e. the dose inducing physiologic levels of EBNA3B) was similar to that seen for the wild-type LCL, whereas higher levels of induction increased recognition accordingly. Assays with different pEBNA3B-tet LCLs, using effector cells against the other four CD8 and two CD4 epitopes in EBNA3B, gave very similar results (data not shown). All subsequent studies were therefore conducted on cells induced to express indicator antigens at physiologic (25 ng/ml dox) and at supra-physiologic (100 ng/ml dox) levels, with similar patterns of results obtained.

### T cell recognition with time after EBNA3B induction

**Figure 2:**
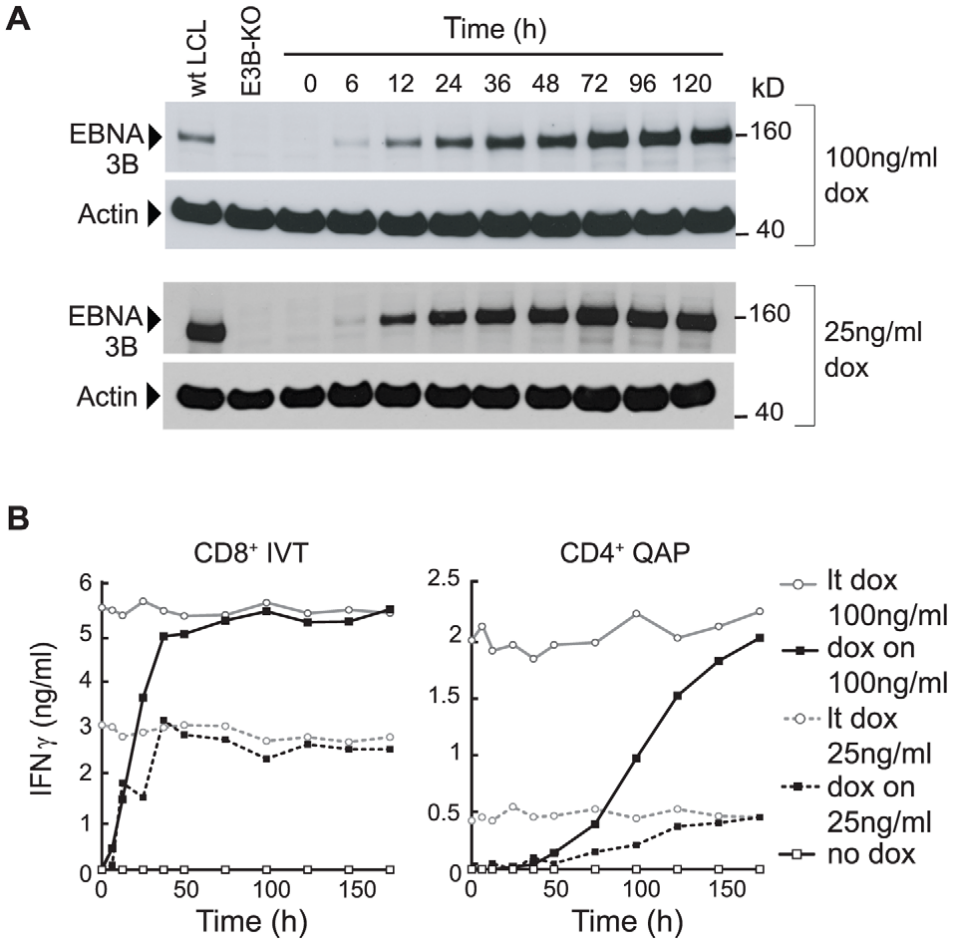
EBNA3B protein expression and T cell recognition of target cells following dox-induction. The pEBNA3B-tet LCL (A*1101, DRB4*01-positive) was induced with 25 ng/ml and 100 ng/ml dox for intervals up to 120 h and (A) analysed for EBNA3B protein by immunoblotting, with wt and EBNA3B-KO LCL (E3B-KO) controls (note the different exposure times used for 25 and 100 ng/ml inductions), and (B) fixed and used as targets, as in Figure 1C, for recognition by CD8^+^ T cells specific for the IVT/A*1101 epitope and by CD4^+^ T cells specific for the ILR/DRB4*01 epitope. Recognition is shown by the black line (dox on); for reference, each assay included non-induced (no dox, open squares) and long-term-induced (lt dox, open circles) cells from the same pEBNA3B-tet LCL. Values are means of triplicate wells with SD always <5%. Similar results were obtained on three occasions.

We first asked how quickly target cells became susceptible to CD8^+^ and CD4^+^ T cell recognition following dox-induction. Figure 2 shows one such experiment inducing the above pEBNA3B-tet LCL at the two dox concentrations. In both cases, expression of EBNA3B protein was detectable by immunoblotting within 6 h of dox addition, and by 72 h had increased to reach a stable steady-state level that was again higher (relative to a wild-type LCL) at the higher inducing dose (Figure 2A). Aliquots of the same cells were used as targets in T cell assays, each time alongside cells from the appropriate non-induced and long-term-induced cultures. To examine epitope display at the precise time of harvest, all target cells were fixed in 1% PFA before addition to the assay. As shown in Figure 2B, while absolute levels of IFNγ release were always higher with targets given 100 ng/ml dox, the same pattern of results was obtained following antigen induction at either dose. Thus, recognition by CD8^+^ T cells specific for the IVT/A*1101 epitope was detectable within 6 h of dox induction and by 36 h had increased to plateau at the same level as seen against long-term dox-induced targets. In contrast, recognition by CD4^+^ T cells specific for the ILR/DRB4*01 epitope was not detectable until 36–48 h and increased quite slowly thereafter, only reaching the long-term dox plateau level on targets induced for 168 h. In further experiments with this and other pEBNA3B-tet LCLs, these temporal differences between CD8 and CD4 epitope display held true for all eight EBNA3B epitopes tested (data not shown).

### T cell recognition with time after EBNA1 induction

**Figure 3:**
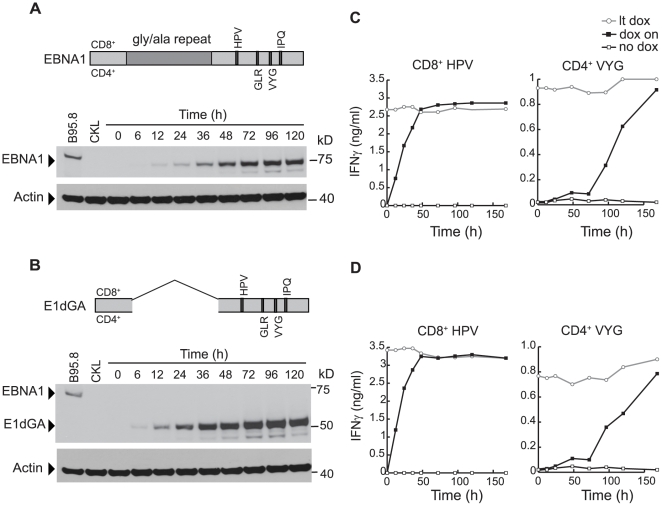
EBNA1 protein expression and T cell recognition of target cells following dox-induction. (A, B) CKL virus-transformed LCLs stably transfected with either (A) the pEBNA1-tet vector encoding a full length B95.8 EBNA1 protein, or (B) the pE1dGA-tet vector encoding a GAr-deleted B95.8 EBNA1 protein; CD8^+^ and CD4^+^ T cell epitopes are shown as in Figure 1A. IH4 immunoblots show protein expression following induction for intervals of up to 120 h with 100 ng/ml dox. B95.8 and CKL virus-transformed LCLs serve as controls; note that CKL virus-coded EBNA1 is not recognised by the IH4 antibody. (C, D) Following dox-induction as above, the pEBNA1-tet- and pE1dGA-tet LCLs (both B*3501, DRB1*11-positive) were fixed and used as targets for CD8^+^ T cells specific for the HPV/B*3501 epitope and by CD4^+^ T cells specific for the VYG/DRB1*11 epitope, as in Figure 1C. Recognition is shown by the black line (dox on); each assay included the appropriate non-induced (no dox, open squares) and long-term-induced (lt dox, open circles) cells. Note that CKL virus-coded EBNA1 is not recognised by HPV-specific CD8^+^ T cells and is barely recognised by VYG-specific CD4^+^ T cells. Values are means of triplicate wells with SD always <5%. Similar results were obtained on three occasions.

The existence of EBNA1 sequence variation between geographically distinct EBV isolates [26] allowed us to generate LCLs using a Chinese virus strain (CKL) with epitope mutations that, for the T cell clones used in these experiments, abrogated CD8 recognition and reduced CD4 recognition to a very low base-line. Into these LCLs, we then introduced an epitope-positive EBNA1 allele under dox-regulated control. As shown in Figure 3, we used both a full length EBNA1 sequence and a sequence (E1dGA) from which the internal glycine-alanine repeat (GAr) domain had been deleted. Note that this GAr domain reportedly offers the wild-type protein some level of protection from CD8^+^ T cell recognition through reducing the rate of its translation from mRNA [30] and/or though stabilising the protein from proteasomal digestion [31]. Figures 3A and B show immunoblots of EBNA1 expression induced in the pEBNA1-tet and pE1dGA-tet LCLs following 100 ng/ml dox induction. As with inducible EBNA3B, the two forms of EBNA1 accumulated to reach their steady state levels by 72–96 h post-induction, though E1dGA was detectable slightly earlier than full length EBNA1 (6 versus 12 h post-induction), and accumulated to slightly higher steady-state levels, a finding consistent with published data [30],[32].

We examined the kinetics of EBNA1 and E1dGA presentation using clones against two CD8^+^ (HPV/B*3501 and IPQ/B*07) and two CD4^+^ (GLR/DQB1*06 and VYG/DRB1*11) T cell epitopes (see Table S1). Results from one such set of assays are shown in Figures 3C and D, using HPV- and VYG-specific effectors and target LCLs established from a B*3501, DRB1*11-positive donor. Focusing first on the CD8^+^ T cell data, we found that both EBNA1 and E1dGA were rapidly recognised by CD8^+^ T cells and reached their plateau values (shown by long-term induced cells) within 48 h. Note that these plateau values were always some 20–30% higher with target cells expressing the E1dGA construct. Given the reported effect of the GAr domain on MHC I processing, we looked in greater detail at early time points in the above experiment, repeating the CD8 assays hourly over the first 12 hr post-induction. As illustrated in Figure S1 for assays conducted with 25 and 100 ng/ml dox inductions, we found that CD8 epitope display from E1dGA was indeed slightly accelerated at early times, typically reaching 35% of its plateau value by 12 hr compared to 25–30% for full length EBNA1. Turning now to the CD4^+^ T cell data in Figures 3C and D, antigen presentation by the MHC II pathway was again profoundly delayed. Thus there was no CD4^+^ T cell recognition of dox-induced target LCLs (other than very weak base-line recognition of the CKL virus-coded EBNA1) until 48 h post-induction, followed by a slow rise that did not reach the long-term plateau value even by 168 h. Both the EBNA1 and E1dGA proteins gave similar results in this respect, although here the plateau level of CD4^+^ T cell recognition was always slightly higher with cells expressing the full length protein. Experiments conducted on a different pair of pEBNA1-tet and pE1dGA-tet LCLs using T cell clones specific for the IPQ/B*07 and GLR/DQB1*06 epitopes gave the same pattern of results (data not shown).

The temporal differences between CD8 and CD4 epitope display therefore held true for all epitopes studied both in EBNA3B and in EBNA1. However we reasoned that the delayed presentation of CD4 epitopes might simply reflect their processing by an indirect route if, as previously shown for EBNA3A and 3C, the source antigens access the MHC II pathway through antigen release and uptake by neighbouring cells in the LCL culture [20]. We first investigated this for EBNA3B by co-cultivating “antigen donor” cells (a pEBNA3B-tet LCL lacking relevant MHC restriction alleles but dox-induced to express cognate antigen) with “antigen-recipient” cells (an antigen-negative EBNA3B-KO LCL with the relevant MHC alleles) for 7 days, then used this mixture as a target for EBNA3B-specific CD4^+^ and CD8^+^ T cell clones. As shown in Figure S2A, we found that co-culture could indeed sensitise recipient cells to recognition by CD4^+^ T cell clones specific for the EBNA3B ILR epitope, although not by the corresponding CD8^+^ IVT clones. However, in parallel experiments where we co-cultured dox-induced pEBNA1-tet “antigen donor” cells with a CKL virus-transformed “antigen recipient” LCL, there was never any recognition of the co-culture by EBNA1-specific CD4^+^ T cells (Figure S2B). Furthermore a second sensitive method of detecting inter-cellular antigen transfer, where recipient cells are fed with 25x-concentrated culture supernatant from donor LCLs [20], again never sensitised recipient cells to EBNA1-specific effectors (Figure S2C). This clearly shows that inter-cellular antigen transfer likely contributes to EBNA3B's presentation via the MHC II pathway in LCL cells; however, as others have also observed [17], endogenously expressed EBNA1 is presented by an intracellular route. Yet, irrespective of these differences, both antigens show delayed presentation via the MHC II pathway following the induction of antigen synthesis.

**Figure 4:**
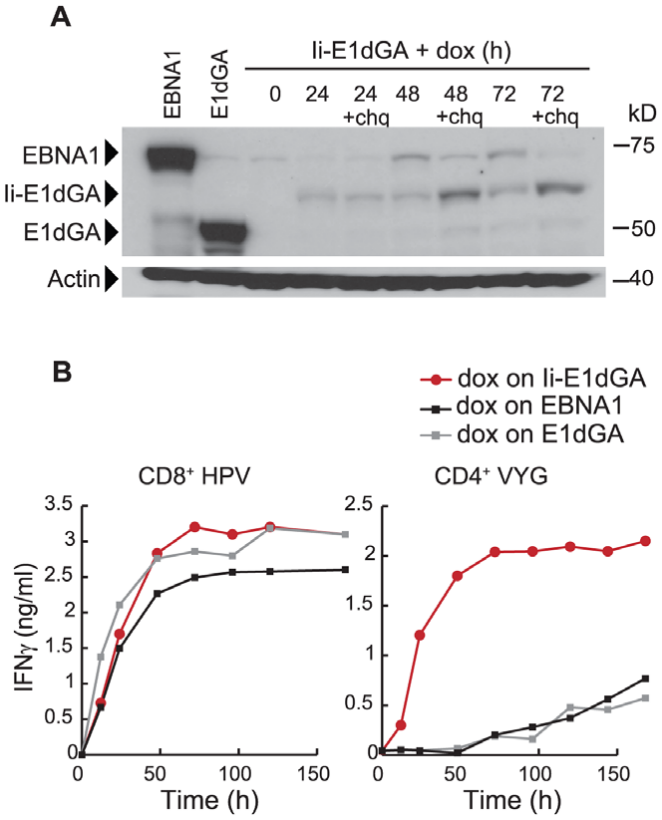
Ii-tagged E1dGA protein expression and T cell recognition of target cells following dox-induction. (A) IH4 immunoblots show EBNA1 protein expression in the pE1dGA-Ii-tet LCL at 24, 48 and 72 h post-induction with 100 ng/ml dox, with or without chloroquine (chq) for the last 24 h before harvest. Controls are the non-induced pE1dGA-Ii-tet LCL (time 0 h) and the pEBNA1-tet and pE1dGA-tet LCLs (as in Figure 3) maintained in long-term dox. (B) Non-induced cultures of the above LCLs (all B*3501, DRB1*11-positive) were induced with 100 ng/ml dox for up to 168 h and assayed for recognition by CD8^+^ T cells specific for the HPV/B*3501 epitope and by CD4^+^ T cells specific for the VYG/DRB1*11 epitope, as in Figure 3C. Results are shown for the pEBNA1-tet LCL (black line), pE1dGA-tet LCL (grey line) and pIi-E1dGA-tet LCL (red line). Values are means of triplicate wells with SD always <5%. Similar results were obtained on three occasions.

We therefore sought reassurance that this slow presentation did not simply reflect an intrinsic feature of MHC class II maturation and epitope display in our LCL cells. To do so, we used the inducible vector system to express E1dGA fused with an invariant chain (Ii) tag that delivers the protein directly into endosomes and the MHC II processing compartment [33]. As shown in Figure 4A, expression of the E1dGA-Ii protein is detectable by immunoblotting 24, 48 and 72 h after 100 ng/ml dox-induction but at very low levels compared to non-tagged EBNA1 and E1dGA. This reflects on-going degradation of the endosomally-targeted E1dGA-Ii protein, since adding chloroquine, an inhibitor of endosomal proteolysis, 24 h prior to harvest increased the level of protein detectable. Figure 4B shows the corresponding T cell assay data following dox-induction. The Ii-tagged protein was rapidly presented not just to CD8^+^ T cells, where it was processed as quickly as the non-targeted constructs, but also to CD4^+^ T cells. In this latter case, recognition appeared within 12 h and became almost maximal by 48 h, much quicker than with the non-tagged proteins. Thus our LCLs can rapidly process and present endogenously expressed antigen, once that antigen gains access to the MHC II presentation pathway.

### T cell recognition with time after down-regulation of antigen synthesis

**Figure 5:**
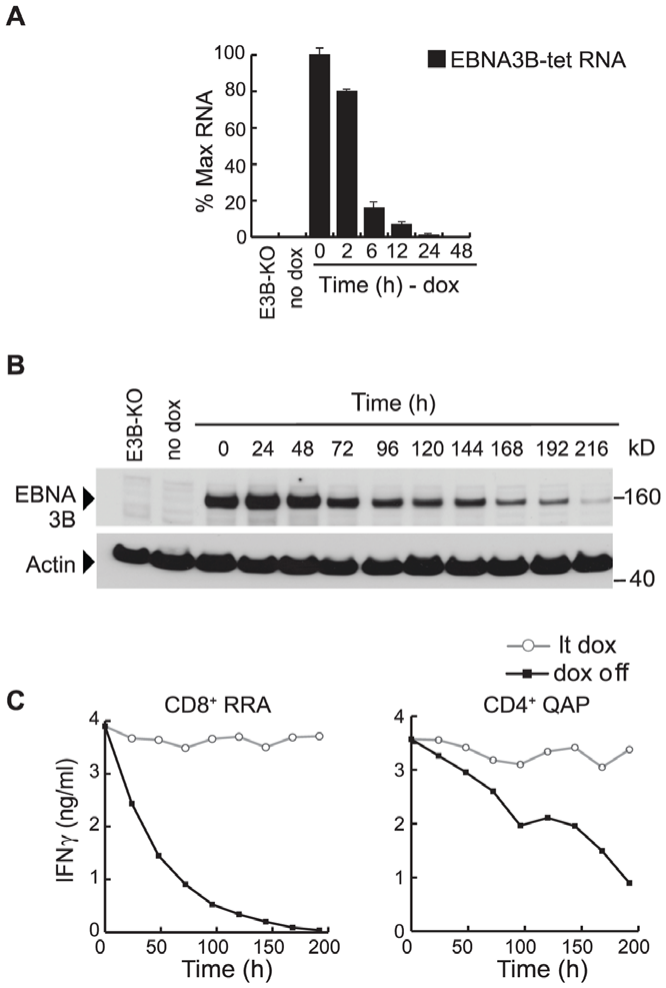
EBNA3B protein expression and T cell recognition of transfected lines following dox-removal. (A) Q-RT-PCR assay of EBNA3B-specific transcript levels (mean of duplicate values +/−SD) in a long-term dox-induced (100 ng/ml) pEBNA3B-tet LCL following dox removal, expressed relative to the level before dox removal. Controls are an EBNA3B-KO virus-transformed LCL (E3B-KO) and the non-induced pEBNA3B-tet LCL (no-dox). (B) Immunoblot showing EBNA3B protein levels in a long-term dox-induced pEBNA3B-tet LCL up to 216 h after dox-removal. Controls are an EBNA3B-KO virus-transformed LCL (E3B-KO) and the non-induced pEBNA3B-tet LCL (no-dox). (C) At different times following removal from 100 ng/ml dox, the above pEBNA3B-tet LCL (B*2702, DRB3*0201-positive) was fixed and assayed for recognition by CD8^+^ T cells specific for the RRA/B*2702 epitope and by CD4^+^ T cells specific for the QAP/DRB3*0201 epitope. Recognition at each time point is shown by the black line. Each assay included long-term-induced cells as a control (lt dox, grey line). Values are means of triplicate wells with SD always <5%. Similar results were obtained on three occasions.

We then examined antigen presentation in long-term 100 ng/ml dox-induced cells after switching off new antigen synthesis by dox-withdrawal. As illustrated in Figure 5A, using a Q-RT-PCR assay for vector-encoded EBNA3B mRNA transcripts, we first showed that >80% of transcripts are lost within 6 h and none are detectable by 24 h. This implies that new antigen synthesis must terminate quite rapidly after dox withdrawal. However, as shown in Figure 5B, the EBNA3B protein is clearly very stable since it remained easily detectable in immunoblots for several days post-withdrawal. Indeed, as the immunoblots were loaded with equal number of cells each time, the falling EBNA3B levels reflect both slow natural turnover of the protein and also dilution from cell doubling (in cultures with a doubling time of 48–72 hr).

Aliquots of LCL cells from the same experiment (HLA B*2702, DRB3*0201-positive) were used in parallel as targets for EBNA3B-specific T cells. As shown in Figure 5C, target cell recognition by an RRA epitope-specific CD8^+^ T cell clone fell progressively after dox withdrawal, down to half of the original level by 48 h, to <10% by 96 h and approaching zero thereafter. By contrast, recognition by a CD4^+^ T cell clone against the QAP epitope fell much more slowly, being still >50% of the original level after 96 h and >20% even after 192 h. Indeed the rate of fall in CD4 epitope display closely paralleled the level of EBNA3B protein detectable in these target cells by immunoblotting (cf. Figures 5B and 5C). Such experiments were conducted on all three pEBNA3B-tet LCL backgrounds, whether first induced at 25 ng/ml or 100 ng/ml dox, and included clones against five CD8 epitopes and three CD4 epitopes. In each case CD8^+^ T cell recognition had fallen to <10% of its original value by 96 h after dox withdrawal, whereas CD4^+^ T cell recognition was still at 35–50% of its original value at the much later time of 168 h (data not shown).

**Figure 6:**
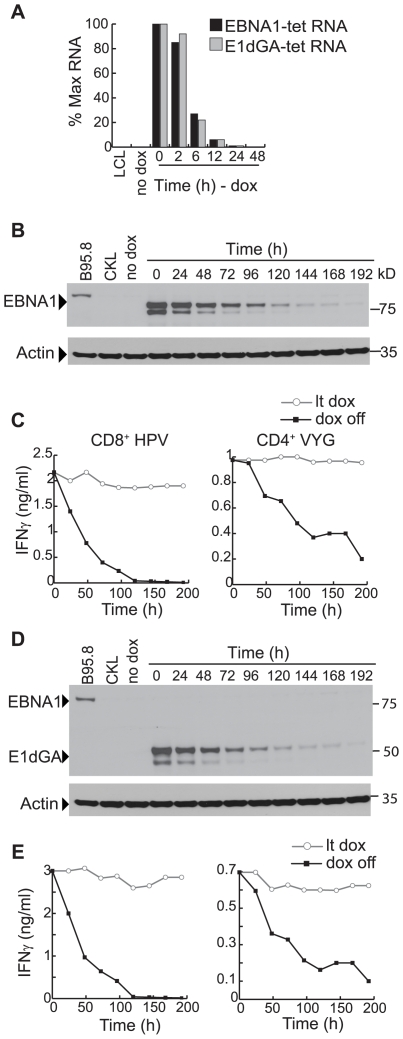
EBNA1 protein expression and T cell recognition of transfected lines following dox removal. (A) Q-RT-PCR assay of vector-expressed EBNA1 and E1dGA transcript levels (mean of duplicate values +/−SD), in long-term dox-induced (100 ng/ml) pEBNA1-tet and pE1dGA-tet LCLs respectively, following the removal of dox; results expressed as in Figure 5A. (B) Immunoblot showing EBNA1 protein levels in a long-term dox-induced pEBNA1-tet LCL up to 192 h following removal from 100 ng/ml dox. Controls are B95.8 and CKL virus-transformed LCLs, and the non-induced pEBNA1-tet LCL. (C) Shown below are results of T cell assays where, at different times following dox-removal, the above pEBNA1-tet LCL (B*3501, DRB1*11-positive) was fixed and assayed for recognition by CD8^+^ T cells specific for the HPV/B*3501 epitope and by CD4^+^ T cells specific for the VYG/DRB1*11 epitope. Recognition is shown by the black line. Each assay included long-term-induced cells as a control (lt dox, grey line). (D, E) Parallel results to those shown in B, C above, using the corresponding pE1dGA-tet LCL. Values are means of triplicate wells with SD always <5%. Similar results were obtained on three occasions.

Results from a corresponding experiment involving pEBNA1-tet and pE1dGA-tet LCLs are shown in Figure 6. Q-RT-PCR assays using primer/probe combinations specific for vector-encoded EBNA1 and E1dGA mRNAs showed mRNA levels fell rapidly after dox-withdrawal and were undetectable beyond 12 h (Figure 6A). Again, therefore, new antigen synthesis must rapidly terminate following dox withdrawal yet, as shown by the immunoblots in Figures 6B and 6D, both the EBNA1 and E1dGA proteins are relatively stable, levels per cell falling slowly over time and being still detectable at 168 h. When these same dox-withdrawn cells (HLA B*3501, DRB1*11-positive) were used as targets in T cell assays, recognition by HPV-specific CD8^+^ T cells fell to <50% of the original level by 48 h and was undetectable by 120 h, whereas recognition by a VYG-specific CD4^+^ T cells fell much more slowly, being still 30–40% of the original value as late as 168 h. Again, parallel experiments using a different LCL background and T cell clones against the other CD8 and CD4 epitopes in EBNA1 produced a very similar pattern of results.

### Persistence of T cell recognition, the half-life of pre-existing epitope complexes and the continued supply of complexes from intracellular sources

**Figure 7:**
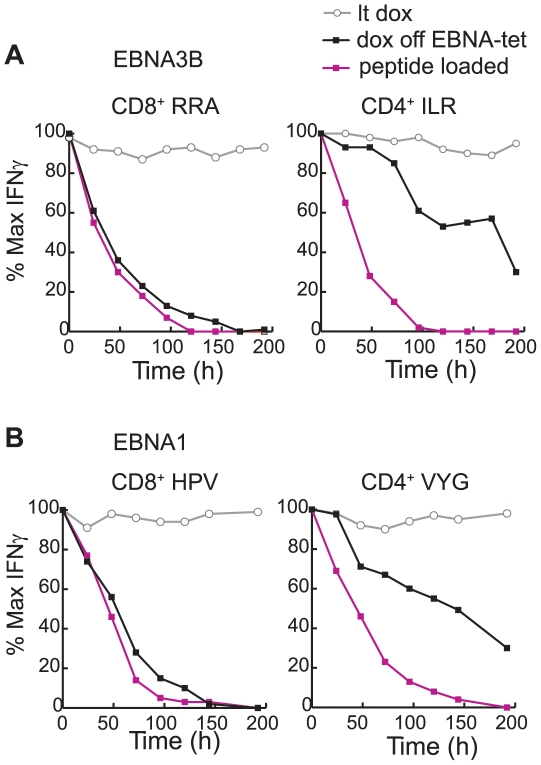
Half-life of MHC-peptide complexes on the cell surface in relation to T cell recognition of transfected lines following dox removal. (A) Results from T cell assays in which an EBNA3B-KO virus-transformed LCL (B*2702, DRB4*01-positive) was loaded with the relevant EBNA3B epitope peptide at a concentration mediating half-maximal recognition, washed well and then used as a target, either immediately (0 h) or up to 192 h later, with CD8^+^ T cells specific for the RRA/B*2702 epitope (left) or CD4^+^ T cells specific for the ILR/DRB4*01 epitope (right). Targets were fixed immediately before the assay. Levels of recognition, expressed as a percentage of that seen at time 0 h, are shown by the red line. In parallel, the same T cells were assayed against a long-term dox-induced pE3B-tet LCL, from the same donor as above, either left in dox throughout the experiment (lt-dox, grey line) or removed from dox at time 0 h (black line). (B) Results from the corresponding experiment using a CKL virus-transformed LCL (B*3501, DRB1*11-positive) loaded with the relevant EBNA1 epitope peptides and assayed using CD8^+^ T cells specific for the HPV/B*3501 epitope (left) or CD4^+^ T cells specific for the VYG/DRB1*11 epitope (right). Results (red line) are shown as above. Parallel assays were carried out on a long-term dox-induced pEBNA1-tet LCL from the same donor, either left in dox (lt-dox, grey line) or dox-depleted at time 0 h (black line) as above. Similar results were obtained on three occasions.

While Figures 5 and 6 showed that CD8 and CD4 epitope display fell at different rates after switching off new antigen synthesis, in both cases target cells remained susceptible to T cell recognition for some time. We therefore asked how the observed rates of fall compared to the half-lives of pre-existing MHC I-peptide and MHC II-peptide complexes on the LCL surface. Thus pEBNA3B-tet and pEBNA1-tet LCLs of the appropriate MHC type maintained in the absence of dox were briefly exposed to a non- saturating dose of epitope peptide, washed well (time 0 h) and the subsequent fall in epitope display tracked over time by T cell assay. For comparison, all experiments included long-term-induced cultures of the same LCLs, from which dox was either withdrawn at time 0 h or maintained throughout. Figure 7 shows representative data obtained for pairs of epitopes from EBNA3B and from EBNA1. Both CD8 epitopes had half-lives on the LCL surface of 36–48 h; indeed the rate with which exogenously loaded CD8 peptides disappeared from the surface was only slightly faster than the rate at which CD8 epitope display fell following cessation of new antigen synthesis. However, both CD4 epitopes also had half-lives in the same range, the levels of display on peptide-pulsed cells therefore falling much quicker than seen on pEBNA3B-tet and pEBNA1-tet LCL cells after cessation of antigen synthesis. A similar pattern of results was observed for all CD8^+^ and CD4^+^ T cell epitopes tested (see for example Figure S3). Such results strongly suggest that, after the cessation of antigen synthesis, new CD4 epitope complexes continue to reach the cell surface whereas the supply of new CD8 epitope complexes is rapidly curtailed.

**Figure 8:**
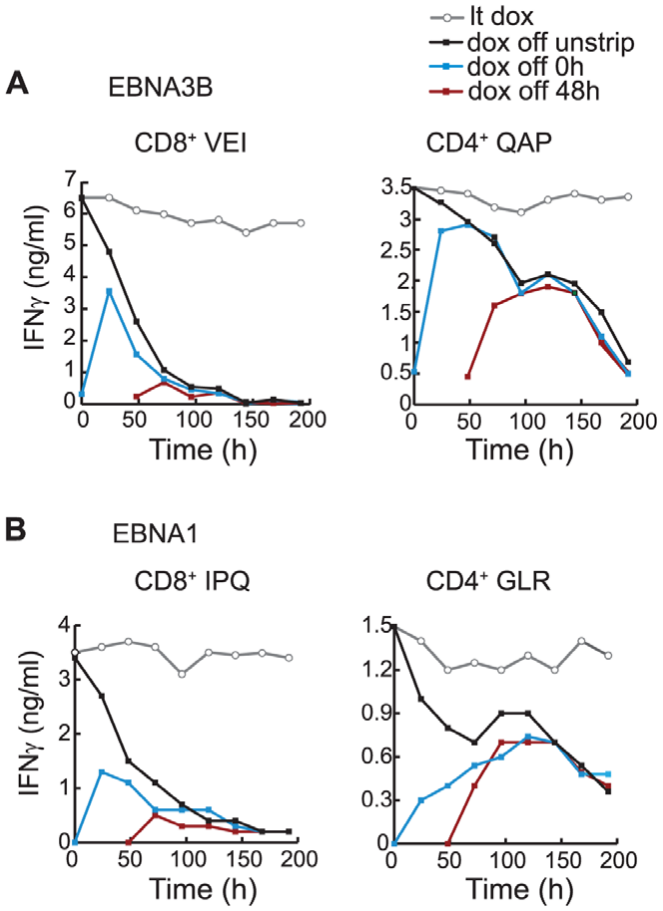
Recovery of T cell recognition of transfected lines following dox removal and stripping of cell surface peptides. (A) Results from T cell assays in which a long-term dox-induced pEBNA3B-tet LCL (B*4402, DRB3*0201-positive) was washed free of dox, and used, either immediately (time 0 h) or up to 192 h later, as targets for a CD8^+^ T cell clone specific for the VEI/B*4402 epitope (left) or a CD4^+^ T cell clone specific for the QAP/DRB3*0201 epitope (right). Results are shown for cells stripped of cell surface peptides either immediately after dox removal (0 h, blue line) or 48 h later (red line), then re-cultured. Targets were fixed immediately before the assay. Results for parallel cultures of non-stripped target cells, either left in dox (lt-dox, grey line) or removed from dox at time 0 h (black line) are shown for comparison. (B) Results from a corresponding experiment using a long-term dox-induced pEBNA1-tet LCL (B*07, DQB1*06-positive) as a target for recognition by a CD8^+^ T cell clone specific for the IPQ/B*07 epitope (left) or a CD4^+^ T cell clone specific for the GLR/DQB1*06 epitope (right). Values are means of triplicate wells with SD always <5%. Similar results were obtained on three occasions.

To test this further, we used a protocol (briefly exposing cells to citrate/phosphate buffer at pH 3.1) that efficiently strips pre-existing EBV epitope/MHC I and/MHC II complexes from the LCL surface without affecting cell viability. Having switched off antigen synthesis in pEBNA3B-tet and pEBNA1-tet LCLs by dox withdrawal, we followed the recovery of epitope peptide display by T cell recognition, stripping pre-existing epitopes off the cell surface either at the time of dox withdrawal (time 0 h) or 48 h later. The results from such assays are illustrated in Figure 8, again comparing CD8/CD4 epitope pairs from EBNA3B and from EBNA1. In each case, new epitope supply after stripping at time 0 h (blue line) or 48 h (red line) is shown against the level of surface epitope display seen on the same target cells that had been similarly dox-depleted at time 0 h but not stripped (black line). The CD8 epitopes showed significant recovery of cell surface display 24 h after stripping at time 0 h but then levels fell away rapidly, down to the same low values remaining on dox-depleted, non-stripped cells. When stripping was delayed until 48 h after dox-withdrawal, there was only a small recovery of CD8 epitope display, recapitulating the low residual values on non-stripped cells. By contrast, the CD4 epitopes showed a substantial recovery whether the cells were stripped at 0 h or 48 h following dox-withdrawal. Furthermore the recovery was sustained for up to 192 h, with stripped cells regaining the same persistent levels of CD4 epitope display as shown by non-stripped cells. Figure S4 shows the results of a similar experiment involving different target LCLs, here initially induced at 25 ng/ml dox, and effectors against different epitopes. This emphasises the point that consistent results were obtained for all CD8/CD4 epitope pairs tested, whether antigen was initially expressed at physiological or supra-physiological levels.

## Discussion

Here we address a generic question regarding pathogens, particularly viruses, that naturally infect target cells in which both the MHC I and MHC II pathways of antigen presentation are active. Antigens endogenously expressed within such an infected cell could potentially be presented by both pathways, rendering the cell visible to CD4^+^ as well as CD8^+^ T cells. However, the relative timing of those events and their degrees of dependence upon new antigen synthesis have never been rigorously examined in parallel. Our experimental system, based on EBV-infected B cell lines and the regulatable expression of EBV antigens, allows one to study these processes in a physiologically relevant cell context, select appropriate levels of antigen expression and track the presentation of CD8 and CD4 epitopes from the same source antigen after inducing or suppressing antigen synthesis. We studied five CD8 and three newly-defined CD4 epitopes from EBNA3B and two CD8 and two CD4 epitopes from EBNA1, in each case probing epitope display with at least two independent clones per epitope. To cover the wide range of MHC restricting alleles involved, assays were conducted on three different pEBNA3B-tet LCLs and two different pairs of pEBNA1-tet and pE1dGA-tet LCLs. The contrasting patterns of CD8 versus CD4 epitope display were remarkably consistent across the whole range of epitopes and antigens studied, and were reproducible whether the antigen was being expressed at physiologic (LCL-like) or supra-physiologic levels. We infer that these differences are not chance consequences of particular epitope selection but reflect fundamental differences in the way that endogenously expressed viral antigens are handled by the MHC I and MHC II presentation pathways in human B cells. At the same time, we would emphasise that both EBNA3B and EBNA1 are native nuclear proteins; there could possibly be differences in detail were one to study the processing of viral antigens normally resident in the cytoplasm or marked for export, but we would nevertheless expect the basic pattern of results to remain the same.

With antigen induction, we found that EBNA3B and both forms of EBNA1 were rapidly recognised by CD8^+^ T cells. Recognition was first apparent soon after dox addition and rose to almost maximal levels within 24 h, well before steady-state levels of these proteins, as detected by immunoblotting, were reached. The results with EBNA1 were particularly interesting given the history of work on this protein as a target for CD8^+^ T cells. Thus early studies found that the GAr domain was able to protect EBNA1 from presentation via the MHC I pathway [31] and that this was associated with resistance to proteasomal degradation [31],[34]. However, more recent results have shown that this protection from CD8^+^ T cell recognition is only partial [35],[36],[37],[38] and may reflect a GAr-mediated reduction of the rate of protein translation rather than of sensitivity to the proteasome *per se* [30],[32],[39],[40]. Importantly, many of these studies involved chimaeric antigen constructs, often with indicator epitope insertions, tested in *in vitro* translation or transient transfection assays, leaving the effects of the GAr domain in its physiologic setting open to question. In the present work we found that, after inducing antigen synthesis, E1dGA was presented to CD8^+^ T cells slightly quicker than the wild-type protein, though the magnitude of the effect was not as great as noted in other less physiologic experimental settings. We believe that our system is robust in this regard since we also found that CD8^+^ T cell recognition of cells induced to express E1dGA long-term was consistently 20–30% greater than seen with cells induced to express EBNA1. This exactly mirrors levels of EBNA1 epitope display seen earlier in LCLs transformed with EBV expressing a GAr-deleted EBNA1 protein versus LCLs transformed with wild-type virus [35].

Overall the results of the antigen induction experiments were consistent with MHC I presentation of newly synthesised polypeptides. However, in the same experiments, the MHC II-restricted presentation of EBNA3B and EBNA1 was grossly delayed; CD4 epitope display only became detectable after 36–48 h and took some 7 days to reach the long-term steady state level. This delay is not an intrinsic feature of MHC II processing in LCL cells since an invariant chain-targeted E1dGA protein expressed in the same dox-inducible system was detected by CD4^+^ T cells within 12 h and maximum recognition was reached within 48 h. This reinforces a large amount of earlier evidence testifying to the efficiency of MHC II antigen processing in LCL cells [41]. Our findings therefore imply that endogenously expressed antigens such as EBNA3B and EBNA1 are delivered very slowly into the MHC II processing pathway, even though they may access that pathway by different routes. Thus co-cultivation experiments showed that EBNA3B (like EBNA3A and 3C, [20]) is processed, at least in part, via the inter-cellular transfer of antigen between LCL cells. The precise form of antigen being transferred in LCL cultures is not known, except that it clearly requires active processing and, by analogy with our earlier work using donor cells transformed with a replication-defective EBV strain [20], does not derive from cells dying as a result of lytic virus replication. By contrast, the same experimental approaches never detected any evidence for inter-cellular transfer of EBNA1. Thus the CD4 epitopes recognised by our EBNA1-specific T cell clones must derive from antigen processed by an intracellular route. In that regard, others have also observed that endogenously expressed EBNA1 is processed intracellularly in LCL cells, and have suggested the involvement of autophagy in that process [17].

Dox-withdrawal from pre-induced LCLs allowed us to ask whether, in the absence of new antigen synthesis, the pre-formed intracellular pool of mature protein can feed the MHC I and MHC II pathways. We first verified that gene transcription from the dox-inducible promoter terminated rapidly after dox-withdrawal, with EBNA3B and EBNA1 transcript levels falling by >80% within 6 h and becoming undetectable by 12-24 h. New antigen synthesis must therefore be curtailed at least at the same rate yet, as is clear from the immunoblots, the pre-formed EBNA3B, EBNA1 and E1dGA proteins remain detectable for days thereafter. In this regard the natural turnover of EBNA3B has not been investigated previously, while ours is the first attempt to compare turnover of the EBNA1 and E1dGA proteins having switched off their synthesis specifically, rather than non-specifically with general protein synthesis inhibitors. Previous studies of the latter kind, where EBNA1 is first expressed by transient transfection or from recombinant viral vectors, all indicate that the wild-type protein has a long half-life, but differ in the degree to which this is shortened by GAr deletion [31],[32],[36],[42]. Our finding, that in the natural setting of the LCL cell both EBNA1 and E1dGA are stable proteins, accords with the most recent findings from transiently transfected cells with protein synthesis inhibitors [42]. For our present purpose, however, the essential point is that both EBNA3B and the two forms of EBNA1 are sufficiently stable that a large pool of mature protein persists in the cells for several days after the cessation of new antigen synthesis, providing a source of antigen that is potentially available to both MHC I and MHC II presentation pathways.

It is therefore significant that, upon dox-withdrawal, T cell assays showed a marked fall in cell surface display of all seven CD8 epitopes tested, typically to <50% of the initial level by 48 h and to <10% by 96 h. Indeed the rate of fall was in each case close to that seen when the corresponding epitope-negative LCL cells were loaded with epitope peptide at non-saturating levels and tracked over time to follow the natural half-life of the MHC I-peptide complex on the cell surface. These half-life measurements accord with earlier work, for example the RRA/B*2702 epitope from EBNA3B was estimated to have a half-life of 40 h in the present T cell assays and of 37 h in earlier antibody-based assays [43]. While rates of fall were similar on dox-depleted and peptide-pulsed cells, there was often a slight delay in the timing of that fall on dox-depleted cells. At least part of this lag must reflect the fact that, for a short time after dox-withdrawal, new MHC-peptide complexes either already in the export pathway or generated from residual mRNA translation will be delivered to the cell surface. Overall, the results strongly suggest that continued CD8 epitope display depends upon continued antigen synthesis. By contrast, T cell recognition of CD4 epitopes consistently fell much more slowly after dox-withdrawal, typically being still >50% of the initial level at 96 h and still easily detectable as late as 192 h. Recognition persisted despite the fact that in peptide pulsing experiments the relevant MHC II/CD4 epitope complexes have half-lives similar to their MHC I/CD8 epitope counterparts, strongly implying that the MHC II presentation pathway was being fed from the mature protein pool.

These conclusions were further supported by experiments in which cells were stripped of cell surface peptides after dox-withdrawal, and then assayed for the recovery of epitope display over time. Interestingly, cells stripped immediately after dox withdrawal showed a significant recovery of detection by CD8^+^ T cells 24 h later; however this effect, which could be quite marked for some epitopes, was transient with recognition falling away at later times. We attribute this transient recovery to the continued supply of newly-formed complexes to the cell surface occurring immediately after dox-withdrawal (as above) and possibly also to the reappearance of pre-existing mature complexes that were recycling from the surface at the time of stripping [44]. Importantly, cells stripped 48 h after dox removal, by which time surface epitope display was declining rapidly, showed minimal recovery of CD8 recognition. This strongly suggests that the mature protein pool, which is still substantial in cells 48 h after dox-withdrawal, makes little if any contribution to the MHC I presentation pathway. By contrast, CD4 epitope display was extensive and prolonged, whether cells were stripped immediately after dox-withdrawal or 48 h later. Such sustained presentation of CD4 epitopes by cells in which *de novo* synthesis of EBNA3B, EBNA1 and E1dGA was terminated 48 h earlier must reflect processing of antigen derived from the mature protein pool.

In summary, we find that in virally-infected human B cells newly-synthesised viral polypeptides, by inference rapidly degraded translation products, are the dominant source of antigen feeding the MHC class I pathway. This does not discount the possibility that the mature protein pool may, in other circumstances or in other cell types, contribute to such a role. Indeed, prompted by a report that irradiation could increase MHC I processing activity in cycloheximide-treated cells [45], we irradiated pEBNA3B-tet and pEBNA1-tet LCLs several days after dox-withdrawal and showed a small, transient recovery of CD8 epitope display that, in the absence of antigen synthesis, must have come from mature protein (L.K. Mackay, unpublished observations). However we find no evidence of any major contribution to the MHC I pathway from this source in a naturally proliferating LCL cell. By contrast, in these same cells endogenous antigen presentation via the MHC II pathway *is* dependent upon the mature protein pool and shows no immediate connection with the presence or absence of *de novo* translation products. These fundamental differences have important implications for virus-specific CD8^+^ and CD4^+^ T cells as direct effectors against infections of MHC I/II-positive target cells. In such circumstances, only CD8^+^ T cells have the capacity to recognise newly infected cells as soon as *de novo* antigen synthesis begins; CD4^+^ T cell recognition will be delayed until the intracellular antigen pool has increased sufficiently to feed the MHC II presentation pathway. Interestingly however, our results imply that for viruses establishing latent infections in MHC I/II-positive cells where viral gene expression is extinguished but where viral proteins persist, a situation that could for example pertain to gamma-herpesviruses and their genome maintenance proteins, the latently-infected cell reservoir may remain visible to CD4^+^ T cells.

## Materials and Methods

### Ethics statement

All experiments were approved by the South Birmingham Local Research Ethics Committee (07/Q2702/24). All patients provided written informed consent for the collection of blood samples and subsequent analysis.

### Stable transfection and establishment of pEBNA-tet cell lines

LCLs were established using the reference EBV strain B95.8, a B95.8-based recombinant lacking the EBNA3B gene (EBNA3B-KO) [23], or the Chinese CKL strain (called NPC 15, [46]) with a variant EBNA1 sequence. All lines were maintained in RPMI 1640 medium supplemented with 2 mM glutamine and 10% fetal calf serum (standard medium). A derivative of the dox-dependent expression vector pRTS-1 [27] and the EBNA3B, EBNA1 and E1dGA constructs were kindly provided by Dr J Mautner, Munich; in cases where EBNA1 was expressed under dox control, a derivative of pRTS-1 lacking constitutively expressed EBNA1 was used. Ii-tagged E1dGA and FLAG-tagged EBNA1 and E1dGA constructs were constructed by PCR, verified by DNA sequencing, then introduced into the vector by standard DNA cloning procedures. To introduce these into LCLs, DNA (15 µg) was transfected into 10^7^ cells by electroporation in 300 µl Optimem (Invitrogen) at 230 V and 960 µF using a Biorad electroporation apparatus. Immediately after electroporation, cells were resuspended in RPMI 10% FCS and were incubated at 37°C and 5% CO_2_. After 24 h in culture, cells were then stained with rat CD2-specific antibody OX34 and were positively selected by magnetic cell sorting with anti-mouse IgG2a/b Microbeads and LS columns (Miltenyi Biotech) according to the manufacturer's guidelines. Cells were then expanded and maintained in culture in the absence of dox, before testing for dox- inducibility of antigen expression.

### Quantitative real-time reverse transcription (Q-RT) PCR

Total RNA was extracted from 5×10^6^ cells using a Nucleospin RNA extraction kit (Macherery Nagel) according to the manufacturer's instructions. 400 ng RNA was reverse transcribed into cDNA using a pool of primers specific for EBNA3B, EBNA1/E1dGA and (as an internal control) cellular GAPDH transcripts. In subsequent quantitative PCR (Q-PCR) assays, primer/probe combinations were used to amplify (i) the 3′ end of the major EBNA3B exon, or (ii) the unique 5′ end of EBNA1/E1dGA transcripts initiated from the dox-regulatable promoter in plasmid pEBNA-tet. After normalising to GAPDH expression, levels of EBNA3B or EBNA1/E1dGA transcription in test cells are expressed relative to that of a fully induced cell line.

### Western blot analysis

Cells were sonicated in UTB buffer (8 M urea, 150 mM β-mercaptoethanol, 50 mM Tris/HCl pH 7.5) and cellular debris removed by centrifugation. Protein concentration was determined by using the BioRad Bradford Protein determination reagent. Solubilized proteins were separated by 8% SDS-PAGE and transferred to nitrocellulose membranes (Thermo Scientific Pierce). Cellular and viral proteins were detected by incubating the membranes with specific Abs followed by HRP-conjugated secondary Abs (Sigma). Bound HRP was visualized using the ECL-plus detection kit (Amersham Biosciences). Antibodies used include: anti-EBNA3B (ExAlpha), anti-EBNA1 (IH4, [47]), and anti-actin (Sigma).

### Epitope-specific T cell clones

CD4 epitope peptides within EBNA3B were identified by screening immune donor lymphocytes in IFNγ Elispot assays on peptide panels (20-mers overlapping by 15) covering the primary sequence of B95.8 strain EBNA3B. All peptides were synthesized using 9-fluorenylmethoxycarbonyl chemistry (Alta Bioscience; University of Birmingham, Birmingham, U.K.), dissolved in DMSO, and concentrations were determined by biuret assay. CD4^+^ and CD8^+^ T cell clones specific for these and for other defined epitopes within EBNA 1 or EBNA3B were generated as described [29]. All epitope sequences are shown, with their MHC restricting alleles, in Table S1.

### Assays of target cell recognition

Immediately before all T cell assays, target LCL cells were fixed in 1% paraformadehyde for 10 min followed by quenching with 0.2 M glycine for 10 min, and then washed with PBS before resuspension in standard medium. Assays therefore measured the level of epitope display at a defined time point, with no further changes occurring during the 18 h assay period itself. Unless otherwise stated, fixed target cells were seeded at 10^5^ cells per triplicate assay well, to which 2000 T cells were added; after 18 h incubation, supernatant medium was harvested and assayed for IFNγ release by ELISA (Endogen). Assays routinely included the following control targets: the wild-type B95.8 virus-transformed LCL from the same donor as the pEBNA-tet-transfected LCLs, the relevant pEBNA-tet-transfected LCL both without dox induction and long-term dox-induced, and the pEBNA-tet-transfected LCL without dox-induction but exogenously loaded with 10^−7^ M concentration of the relevant epitope peptide. In all assays, at least two different T cell clones were tested for each epitope specificity.

### Peptide half-life and peptide stripping assays

In assays measuring the half-life of peptide/MHC complexes at the cell surface, LCLs with relevant HLA types but transformed with EBNA3B-KO or CKL (variant EBNA1) virus strains were exposed for 1 h to epitope peptide at concentrations mediating half-maximal recognition, then washed several times and either fixed immediately for T cell assay, or cultured in standard medium then harvested and fixed for assay at later times. For assays measuring the continued supply of complexes to the surface from intracellular sources, we used an acid-stripping protocol that preliminary work confirmed would completely remove both MHC I- and MHC II-bound epitope peptides without affecting cell viability ([35] and L. Mackay, unpublished observations). Cells were washed with PBS and pellets were gently resuspended in citrate/phosphate buffer (0.131 M citric acid, 0.066 M Na_2_HPO_4_), pH 3.1, for 20 min on ice before neutralization by addition of excess standard medium. Stripped target cells were then washed several times and an aliquot of cells fixed immediately for T cell assay, while the remaining cells were re-cultured in standard medium, then harvested and fixed for assay at later times.

## Supporting Information

Table S1 T cell epitopes used as indices of antigen presentation
(0.01 MB PDF)

Figure S1 CD8^+^ T cell recognition of EBNA1 and E1dGA early post-induction. Results of a similar experiment to those shown in Figure 3 but focusing on CD8 epitope presentation in the first 12 h following induction of pEBNA1-tet- and pE1dGA-tet LCLs (B*3501-positive) with dox at 25 ng/ml (left panels) and 100 ng/ml dox (right panels). Cells, harvested at hourly intervals, were fixed and assayed for recognition by HPV epitope-specific CD8^+^ T cells. (A) Levels of recognition, expressed as IFNγ release, are shown for the pEBNA1-tet (black line) and pE1dGA-tet (blue line) LCLs; each assay included the appropriate long-term dox-induced LCL as a control (lt dox, open circles). Values are means of triplicate wells with SD always <5%. (B) Levels of recognition of the above targets, now expressed as a percentage of maximum IFNγ release seen with the appropriate long-term dox-induced LCL. Similar results were obtained on two occasions.
(0.06 MB PDF)

Figure S2 T cell recognition assays involving target cell mixtures. (A) Assays conducted using T cell clones specific for the CD8 epitope IVT/A*1101 and for the CD4 epitope ILR/DRB4*01, both in EBNA3B. A dox-induced pEBNA3B-tet “donor” LCL [D] expressing EBNA3B but A*1101, DRB4*01-negative was co-cultured 1:1 for 7 days with a “recipient” EBNA3B-KO virus-transformed LCL [R] which was A*1101, DRB4*01-positive, producing the target cell mixture [D+R]. The D and R lines cultured separately served as negative control targets, the D+R co-culture pre-exposed to the epitope peptides immediately before the T cell assay served as a positive control target. (B) Similar assays conducted using T cell clones specific for the CD8 epitope HPV/B*3501 and for the CD4 epitope VYG/DRB*11, both in EBNA1. Here a dox-induced pEBNA1-tet LCL lacking the B*3501 and DRB*11 alleles was the donor, and a CKL virus-transformed LCL positive for the B*3501 and DRB*11 alleles was the recipient. Recognition (mean of triplicate values +/− SD) is expressed as IFNγ units/ml detected in assay supernatant by ELISA. Similar patterns of results were obtained on three occasions, and also using a pE1dGA-tet LCL as the donor. (C) Culture supernatants were harvested from 4 day-old cultures of a B95.8 virus-transformed MHC mis-matched LCL (expressing cognate EBNA1) and, as a control, of the EBV-negative B lymphoma cell line BJAB, both grown in serum-free AIM-V medium as described [20]. Supernatants were concentrated 25-fold and then added to a DRB1*11-positive CKL virus-transformed LCL. After overnight incubation, supernatant-exposed (and untreated cells as a control) were washed and used as targets for recognition by CD4^+^ T cells specific for the EBNA1-derived DRB1*11/VYG epitope. MHC-matched (DRB1*11-positive) and MHC mis-matched (DRB1*11-negative) B95.8 virus-transformed LCLs served as positive and negative control targets respectively. Results are expressed as above.
(0.07 MB PDF)

Figure S3 Half-life of MHC-peptide complexes on the cell surface in relation to T cell recognition of transfected lines following dox removal. Representative results from further peptide pulsing experiments of the kind shown in Figure 7, now using (A) a different E3B-KO virus-transformed LCL (A*1101, DRB3*0201-positive) loaded with the relevant epitope peptides, then washed and used as targets up to 192h later with T cells specific for the AVF/A*1101 CD8 epitope and the QAP/DRB3*0201 CD4 epitope, both from EBNA3B, and (B) a different CKL virus-transformed LCL (B*07, DQB1*06-positive) peptide loaded and washed as above, then used as targets for T cells specific for the IPQ/B*07 CD8 epitope and for the GLR/DQB1*06 CD4 epitope, both from EBNA1. Experimental controls included and expression of results is as described in Figure 7. Values are means of triplicate wells with SD always <5%. Similar results were obtained on three occasions.
(0.07 MB PDF)

Figure S4 Recovery of T cell recognition of transfected lines following dox removal and stripping of cell surface peptides. Representative results from a peptide stripping experiment of the kind shown in Figure 8, using target LCLs removed from 25 ng/ml dox. This experiment used (A) a different long-term dox-induced pE3B-tet LCL (A*1101, ILR/DRB4*01-positive) washed free of dox and used, either immediately (time 0 h) or up to 192 h later, as a target for recognition by T cells specific for the IVT/A*1101 CD8 epitope and the ILR/DRB4*01 CD4 epitope, both from EBNA3B, and (B) a different long-term dox-induced pEBNA1-tet LCL (B*3501, DRB1*11-positive) washed free of dox as above and assayed with T cells specific for the HPV/B*3501 CD8 epitope and the VYG/DRB1*11 CD4 epitope, both from EBNA1. Results are shown for cells stripped of cell surface peptides either immediately after dox removal (0 h, blue line) or 48 h later (red line), then re-cultured. Targets were fixed immediately before the assay. Results for parallel cultures of non-stripped target cells, either left in dox (lt-dox, grey line) or removed from dox at time 0 h as above (black line) are shown for comparison. Values are means of triplicate wells with SD always <5%. Similar results were obtained on three occasions.
(0.12 MB PDF)
