# Supplementary material for: T Cell Detection of a B-Cell Tropic Virus Infection: Newly-Synthesised versus Mature Viral Proteins as Antigen Sources for CD4 and CD8 Epitope Display
Source: PLoS Pathog. 2009 Dec 18;5(12):e1000699. doi: 10.1371/journal.ppat.1000699 (PMC2788701; doi:10.1371/journal.ppat.1000699)
Supplement: Table S1 — T cell epitopes used as indices of antigen presentation (0.01 MB PDF) [file ppat.1000699.s001.pdf]

Table S1. T cell epitopes used as indices of antigen presentation

Antigen	T cell	Epitope coordinates	Epitope sequence	MHC restriction
EBNA3B	$CD8^+$	149-157	HRCQAIRKK	B*2705
		244-254	RRARSLSAERY	B*2702
		399-408	AVFDRKSDAK	A*1101
		416-424	IVTDFSVIK	A*1101
		657-666	VEITPYKPTW	B*4402
	$CD4^+$	281-300	FIEFVGWLCKKDHTHIREWF	DRB1*1501
		771-790	ILRQLLTGGVKKGRPSLKLQ	DRB4*01
		846-865	QAPTEYTRERRGVGPMPPT	DRB3*0201
EBNA1	$CD8^+$	407–417	HPVGEADYFEY	B*3501
		528-536	IPQCRLTPL	B*07
	$CD4^+$	484-503	GLRALLARSHVERTTDEGTW	DQB1*06
		509-528	VYGGSKTSLYNLRRGTALAI	DRB1*11
