# Supplementary figures and images for: T Cell Detection of a B-Cell Tropic Virus Infection: Newly-Synthesised versus Mature Viral Proteins as Antigen Sources for CD4 and CD8 Epitope Display
Source: PLoS Pathog. 2009 Dec 18;5(12):e1000699. doi: 10.1371/journal.ppat.1000699 (PMC2788701; doi:10.1371/journal.ppat.1000699)

**A**

25ng/ml dox

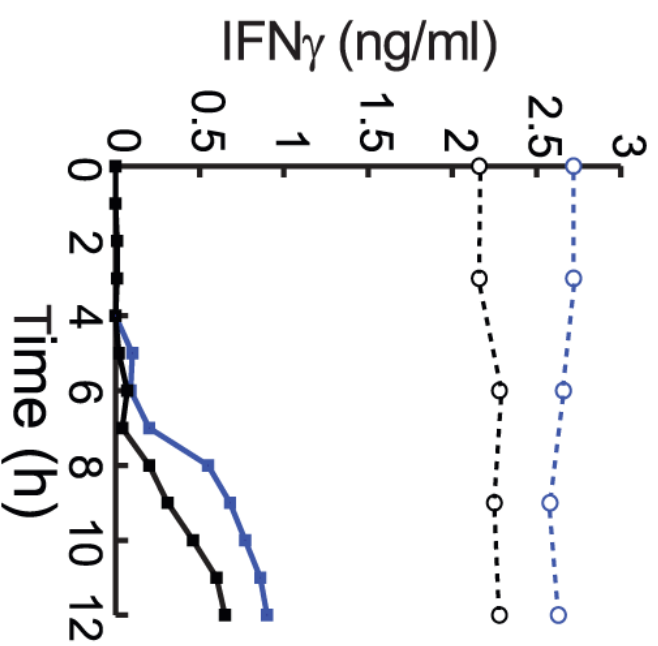

100ng/ml dox

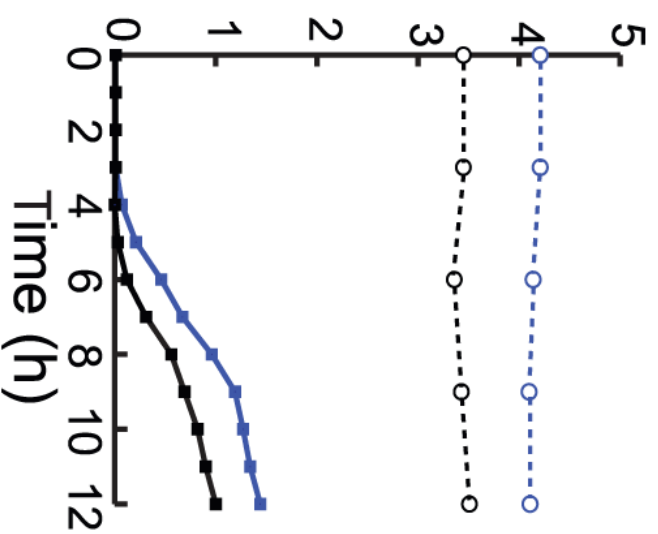**B**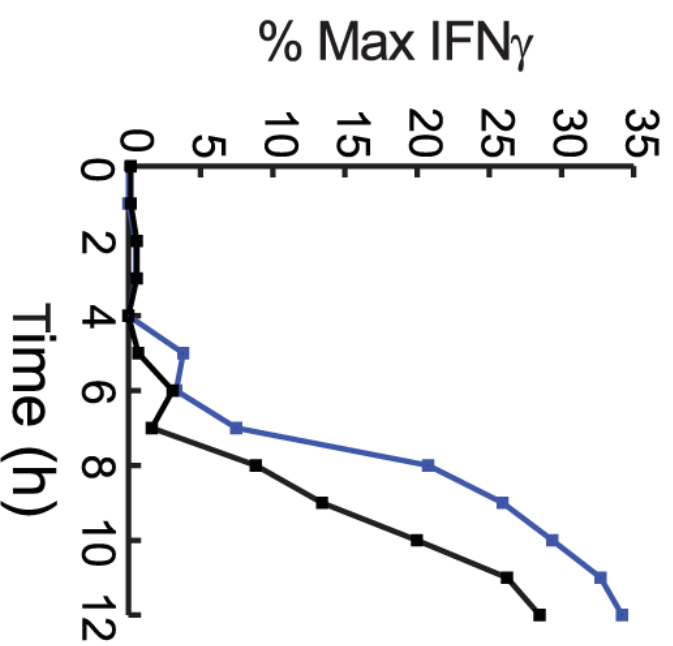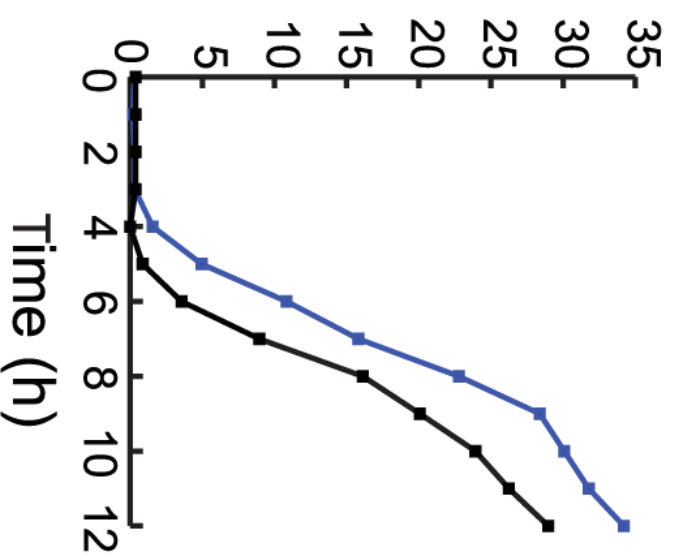

--○-- It dox E1dGA  
--○-- It dox EBNA1  
—■— dox on E1dGA  
—■— dox on EBNA1

Supplement: Figure S1 — CD8+ T cell recognition of EBNA1 and E1dGA early post-induction. Results of a similar experiment to those shown in Figure 3 but focusing on CD8 epitope presentation in the first 12 h following induction of pEBNA1-tet- and pE1dGA-tet LCLs (B*3501-positive) with dox at 25 ng/ml (left panels) and 100 ng/ml dox (right panels). Cells, harvested at hourly intervals, were fixed and assayed for recognition by HPV epitope-specific CD8+ T cells. (A) Levels of recognition, expressed as IFNγ release, are shown for the pEBNA1-tet (black line) and pE1dGA-tet (blue line) LCLs; each assay included the appropriate long-term dox-induced LCL as a control (lt dox, open circles). Values are means of triplicate wells with SD always <5%. (B) Levels of recognition of the above targets, now expressed as a percentage of maximum IFNγ release seen with the appropriate long-term dox-induced LCL. Similar results were obtained on two occasions. (0.06 MB PDF) [file ppat.1000699.s002.pdf]

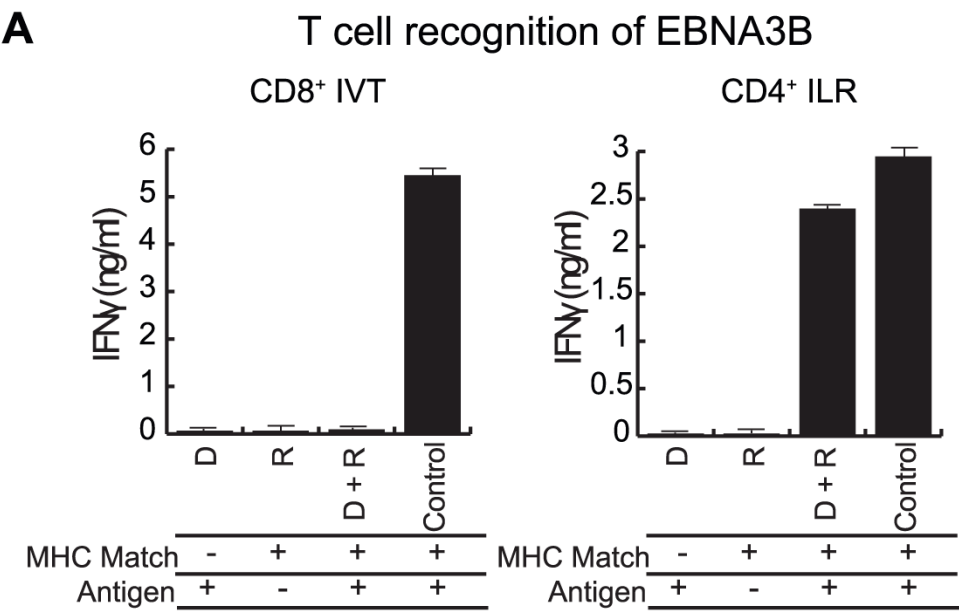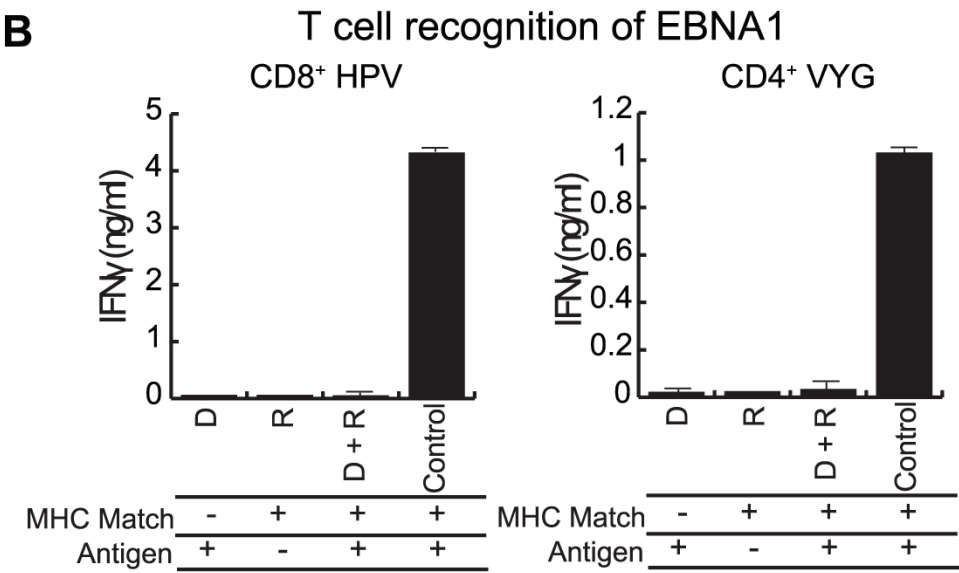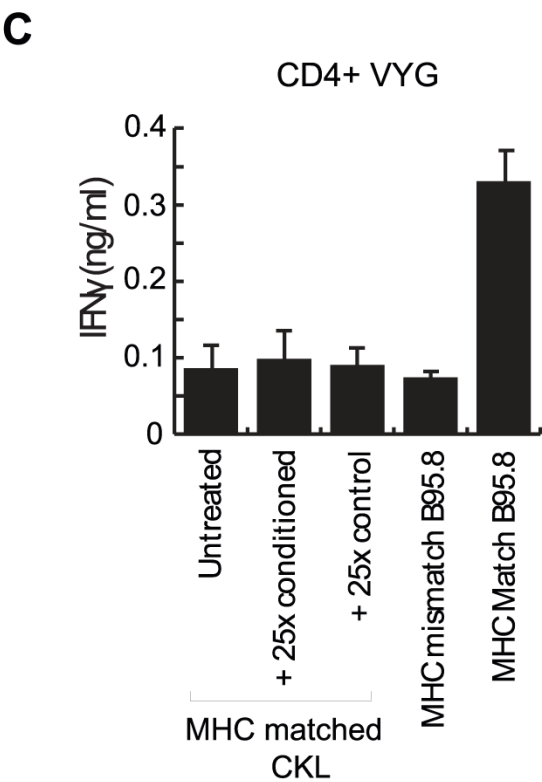

Supplement: Figure S2 — T cell recognition assays involving target cell mixtures. (A) Assays conducted using T cell clones specific for the CD8 epitope IVT/A*1101 and for the CD4 epitope ILR/DRB4*01, both in EBNA3B. A dox-induced pEBNA3B-tet “donor” LCL [D] expressing EBNA3B but A*1101, DRB4*01-negative was co-cultured 1:1 for 7 days with a “recipient” EBNA3B-KO virus-transformed LCL [R] which was A*1101, DRB4*01-positive, producing the target cell mixture [D+R]. The D and R lines cultured separately served as negative control targets, the D+R co-culture pre-exposed to the epitope peptides immediately before the T cell assay served as a positive control target. (B) Similar assays conducted using T cell clones specific for the CD8 epitope HPV/B*3501 and for the CD4 epitope VYG/DRB*11, both in EBNA1. Here a dox-induced pEBNA1-tet LCL lacking the B*3501 and DRB*11 alleles was the donor, and a CKL virus-transformed LCL positive for the B*3501 and DRB*11 alleles was the recipient. Recognition (mean of triplicate values +/− SD) is expressed as IFNγ units/ml detected in assay supernatant by ELISA. Similar patterns of results were obtained on three occasions, and also using a pE1dGA-tet LCL as the donor. (C) Culture supernatants were harvested from 4 day-old cultures of a B95.8 virus-transformed MHC mis-matched LCL (expressing cognate EBNA1) and, as a control, of the EBV-negative B lymphoma cell line BJAB, both grown in serum-free AIM-V medium as described [20]. Supernatants were concentrated 25-fold and then added to a DRB1*11-positive CKL virus-transformed LCL. After overnight incubation, supernatant-exposed (and untreated cells as a control) were washed and used as targets for recognition by CD4+ T cells specific for the EBNA1-derived DRB1*11/VYG epitope. MHC-matched (DRB1*11-positive) and MHC mis-matched (DRB1*11-negative) B95.8 virus-transformed LCLs served as positive and negative control targets respectively. Results are expressed as above. (0.07 MB PDF) [file ppat.1000699.s003.pdf]

**A****EBNA3B****CD8<sup>+</sup> AVF**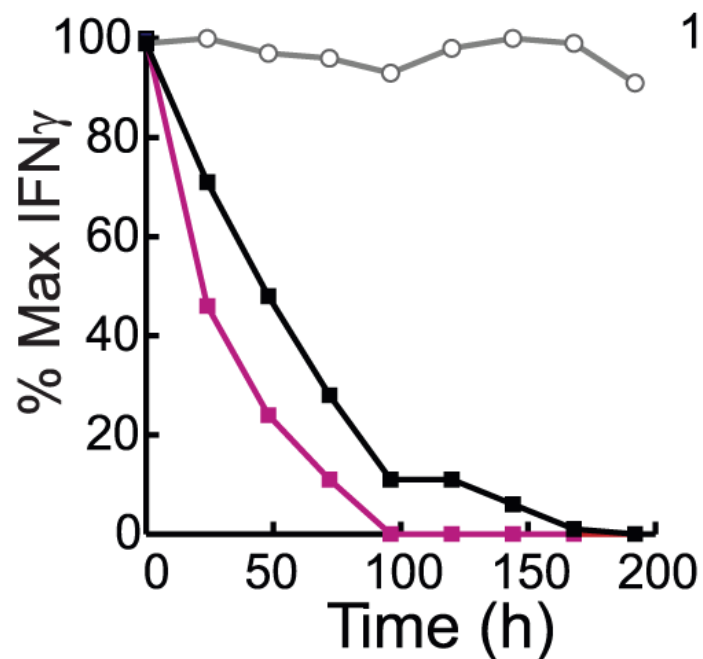**CD4<sup>+</sup> QAP**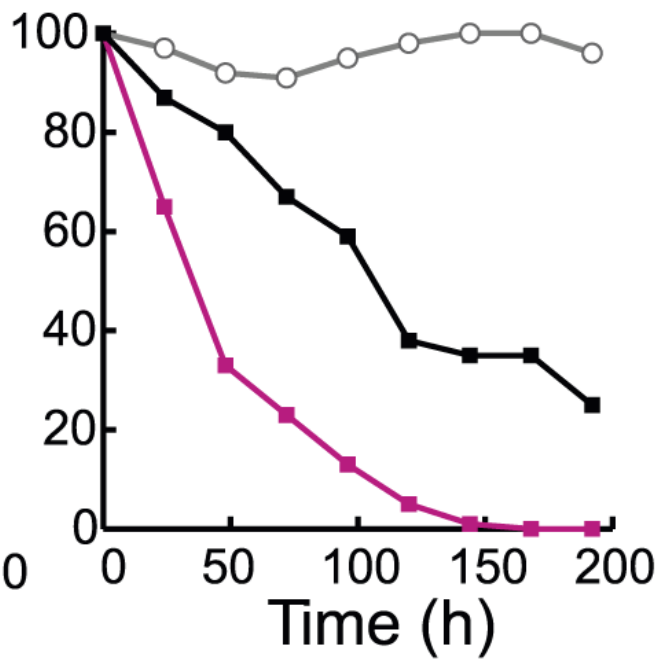**B****EBNA1****CD8<sup>+</sup> IPQ**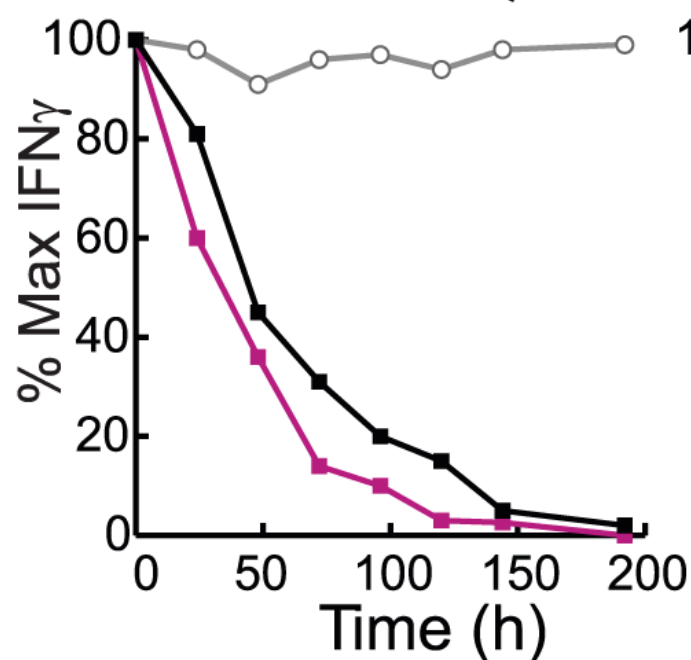**CD4<sup>+</sup> GLR**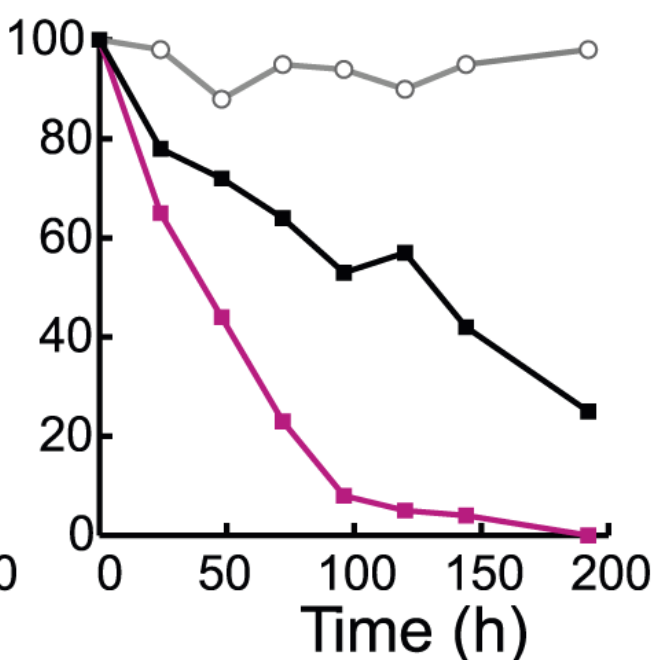

Supplement: Figure S3 — Half-life of MHC-peptide complexes on the cell surface in relation to T cell recognition of transfected lines following dox removal. Representative results from further peptide pulsing experiments of the kind shown in Figure 7, now using (A) a different E3B-KO virus-transformed LCL (A*1101, DRB3*0201-positive) loaded with the relevant epitope peptides, then washed and used as targets up to 192h later with T cells specific for the AVF/A*1101 CD8 epitope and the QAP/DRB3*0201 CD4 epitope, both from EBNA3B, and (B) a different CKL virus-transformed LCL (B*07, DQB1*06-positive) peptide loaded and washed as above, then used as targets for T cells specific for the IPQ/B*07 CD8 epitope and for the GLR/DQB1*06 CD4 epitope, both from EBNA1. Experimental controls included and expression of results is as described in Figure 7. Values are means of triplicate wells with SD always <5%. Similar results were obtained on three occasions. (0.07 MB PDF) [file ppat.1000699.s004.pdf]

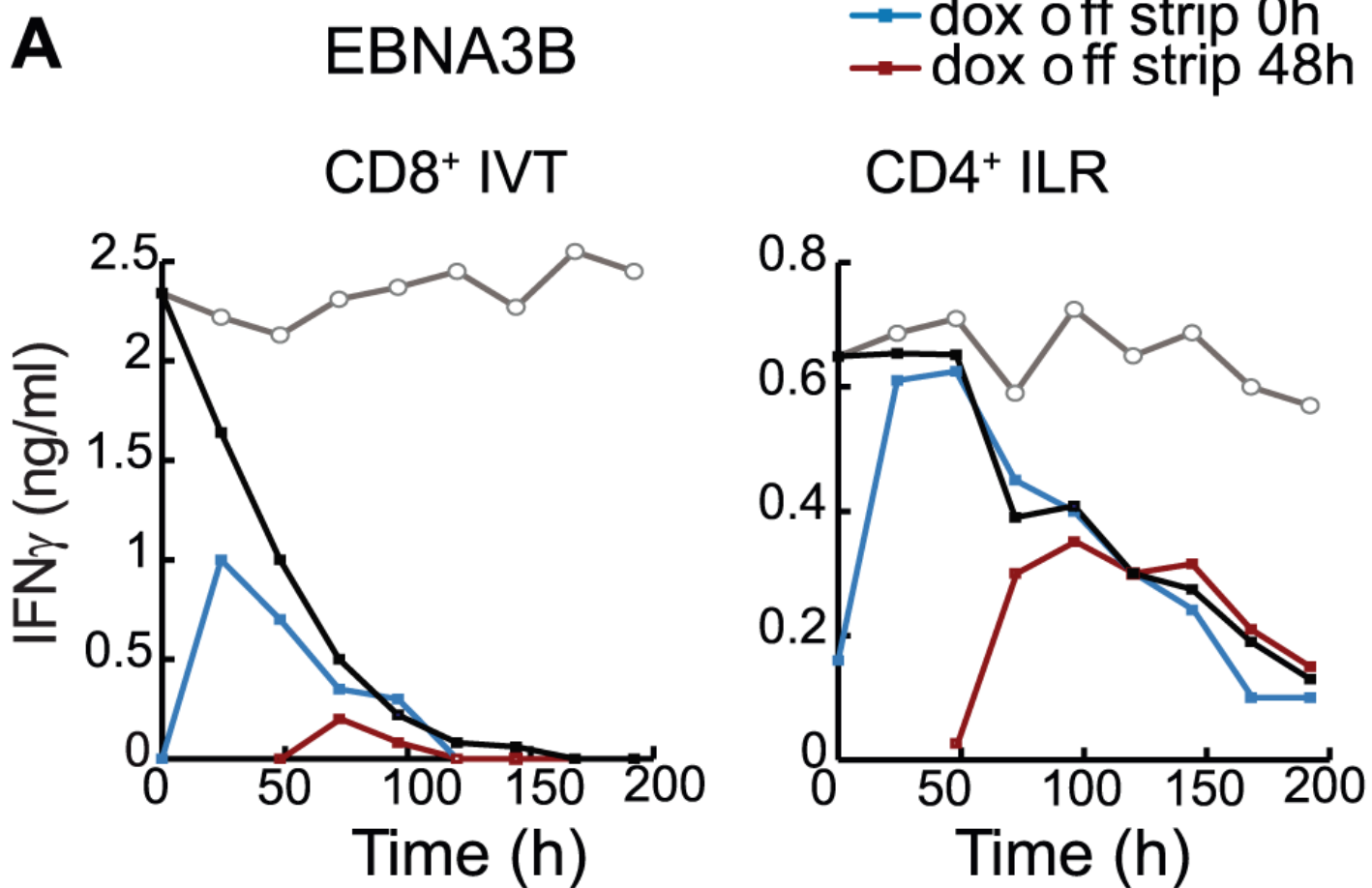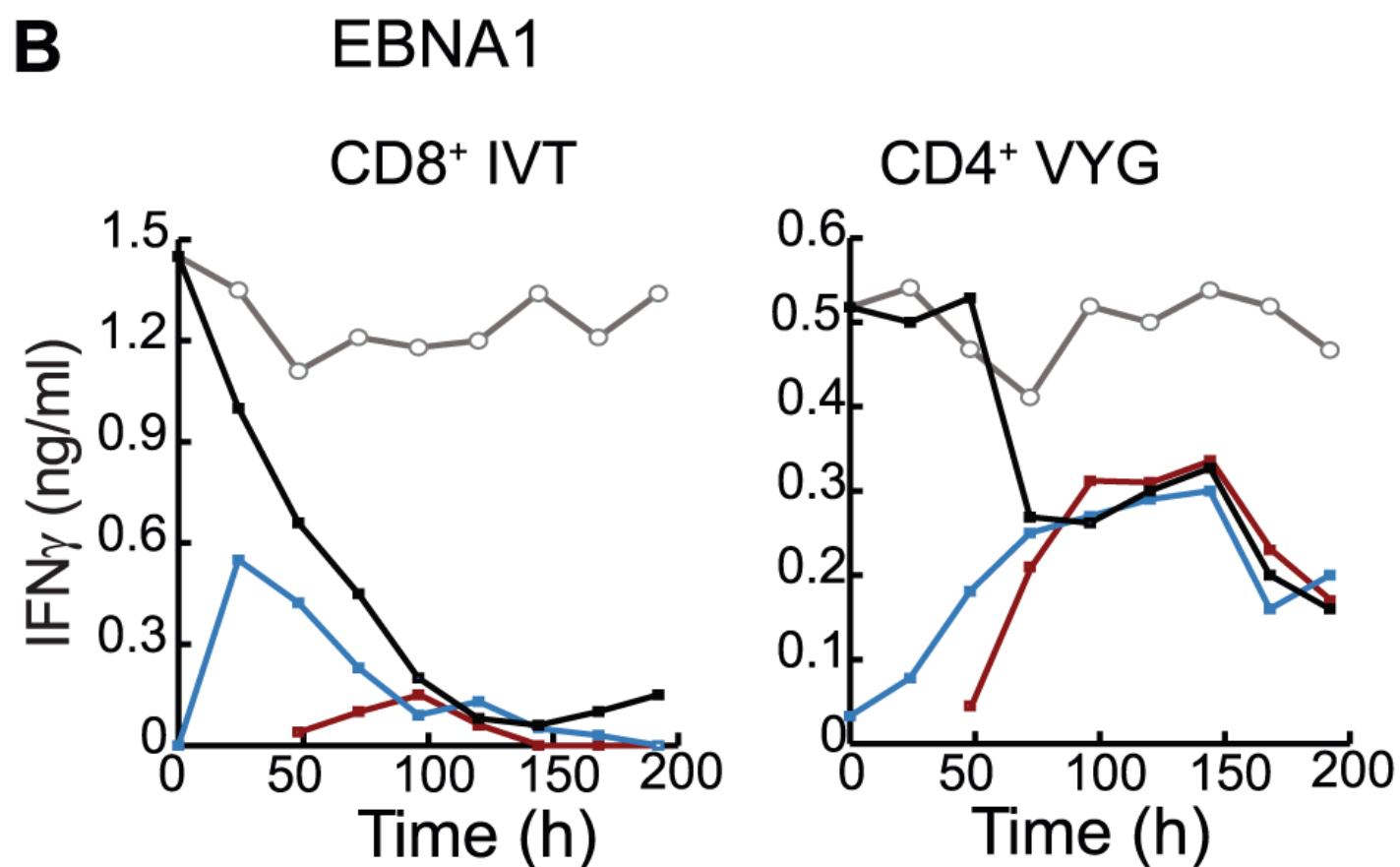

Supplement: Figure S4 — Recovery of T cell recognition of transfected lines following dox removal and stripping of cell surface peptides. Representative results from a peptide stripping experiment of the kind shown in Figure 8, using target LCLs removed from 25 ng/ml dox. This experiment used (A) a different long-term dox-induced pE3B-tet LCL (A*1101, ILR/DRB4*01-positive) washed free of dox and used, either immediately (time 0 h) or up to 192 h later, as a target for recognition by T cells specific for the IVT/A*1101 CD8 epitope and the ILR/DRB4*01 CD4 epitope, both from EBNA3B, and (B) a different long-term dox-induced pEBNA1-tet LCL (B*3501, DRB1*11-positive) washed free of dox as above and assayed with T cells specific for the HPV/B*3501 CD8 epitope and the VYG/DRB1*11 CD4 epitope, both from EBNA1. Results are shown for cells stripped of cell surface peptides either immediately after dox removal (0 h, blue line) or 48 h later (red line), then re-cultured. Targets were fixed immediately before the assay. Results for parallel cultures of non-stripped target cells, either left in dox (lt-dox, grey line) or removed from dox at time 0 h as above (black line) are shown for comparison. Values are means of triplicate wells with SD always <5%. Similar results were obtained on three occasions. (0.12 MB PDF) [file ppat.1000699.s005.pdf]
